# Targeting of mitochondrial fission through natural flavanones elicits anti-myeloma activity

**DOI:** 10.1186/s12967-024-05013-0

**Published:** 2024-02-27

**Authors:** Roberta Torcasio, Maria Eugenia Gallo Cantafio, Claudia Veneziano, Carmela De Marco, Ludovica Ganino, Ilenia Valentino, Maria Antonietta Occhiuzzi, Ida Daniela Perrotta, Teresa Mancuso, Filomena Conforti, Bruno Rizzuti, Enrica Antonia Martino, Massimo Gentile, Antonino Neri, Giuseppe Viglietto, Fedora Grande, Nicola Amodio

**Affiliations:** 1 Department of Experimental and Clinical Medicine, University Magna Graecia of Catanzaro, Viale Europa, Campus Germaneto, 88100 Catanzaro, Italy; 2 Department of Biology, Ecology and Earth Sciences, University of Calabria, Cosenza, Italy; 3 Department of Pharmacy, Health and Nutritional Sciences, University of Calabria, 87036 Rende, CS Italy; 4 Department of Biology, Ecology and Earth Sciences, Centre for Microscopy and Microanalysis, University of Calabria, Cosenza, Italy; 5 Annunziata” Regional Hospital Cosenza, 87100 Cosenza, Italy; 6 SS Rende (CS), Department of Physics, CNR-NANOTEC, University of Calabria, Via Pietro Bucci, 87036 Rende, CS Italy; 7 Institute for Biocomputation and Physics of Complex Systems (BIFI), Joint Unit GBsC-CSIC-BIFI, University of Zaragoza, 50018 Saragossa, Spain; 8 Scientific Directorate, IRCCS Di Reggio Emilia, Emilia Romagna, Reggio Emilia, Italy

**Keywords:** Flavanones, Hesperitin, Naringenin, Mitochondrial dynamics, Multiple myeloma

## Abstract

**Background:**

Mitochondrial alterations, often dependent on unbalanced mitochondrial dynamics, feature in the pathobiology of human cancers, including multiple myeloma (MM). Flavanones are natural flavonoids endowed with mitochondrial targeting activities. Herein, we investigated the capability of Hesperetin (Hes) and Naringenin (Nar), two aglycones of Hesperidin and Naringin flavanone glycosides, to selectively target Drp1, a pivotal regulator of mitochondrial dynamics, prompting anti-MM activity.

**Methods:**

Molecular docking analyses were performed on the crystallographic structure of Dynamin-1-like protein (Drp1), using Hes and Nar molecular structures. Cell viability and apoptosis were assessed in MM cell lines, or in co-culture systems with primary bone marrow stromal cells, using Cell Titer Glo and Annexin V-7AAD staining, respectively; clonogenicity was determined using methylcellulose colony assays. Transcriptomic analyses were carried out using the Ion AmpliSeq™ platform; mRNA and protein expression levels were determined by quantitative RT-PCR and western blotting, respectively. Mitochondrial architecture was assessed by transmission electron microscopy. Real time measurement of oxygen consumption was performed by high resolution respirometry in living cells. In vivo anti-tumor activity was evaluated in NOD-SCID mice subcutaneously engrafted with MM cells.

**Results:**

Hes and Nar were found to accommodate within the GTPase binding site of Drp1, and to inhibit Drp1 expression and activity, leading to hyperfused mitochondria with reduced OXPHOS. In vitro, Hes and Nar reduced MM clonogenicity and viability, even in the presence of patient-derived bone marrow stromal cells, triggering ER stress and apoptosis. Interestingly, Hes and Nar rewired MM cell metabolism through the down-regulation of master transcriptional activators (SREBF-1, c-MYC) of lipogenesis genes. An extract of Tacle, a Citrus variety rich in Hesperidin and Naringin, was capable to recapitulate the phenotypic and molecular perturbations of each flavanone, triggering anti-MM activity in vivo.

**Conclusion:**

Hes and Nar inhibit proliferation, rewire the metabolism and induce apoptosis of MM cells via antagonism of the mitochondrial fission driver Drp1. These results provide a framework for the development of natural anti-MM therapeutics targeting aberrant mitochondrial dependencies.

**Supplementary Information:**

The online version contains supplementary material available at 10.1186/s12967-024-05013-0.

## Introduction

Multiple myeloma (MM) is a complex and incurable disease, accounting for approximately 1% of all cancers and 10% of all haematological malignancies. Its defining feature is the clonal proliferation of malignant plasma cells in the bone marrow (BM), leading, among other complications, to bone damage, weakened immune function, and kidney dysfunction [1]. Despite substantial therapeutic advancements in the last decade, understanding the pathobiology of MM and developing new treatments remain a challenge, especially in the chemo-resistant advanced stages [2].

Mitochondria are organelles essential for various cellular processes, including the synthesis of ATP and the production of ROS and metabolites; they also participate in the regulation of several cell death pathways, as well as in the onset of endoplasmic reticulum (ER) stress and the integrated stress response (ISR) [3].

In recent years, mitochondria have emerged as crucial players in several cancer types, including MM, in which malignant plasma cells (PC) progressively acquire defects in mitochondrial functions, including the increase in mitochondrial mass, alterations in mitochondrial DNA, and changes in mitochondrial morphology, leading to impaired bioenergetics and metabolism [4, 5].

Mitochondria constantly undergo processes, such as fission (division), fusion (merging) and movement inside cells, regulated by GTPase proteins. Fusion is the merging of two separate mitochondria into one, facilitating content exchange and maintaining mitochondrial health; conversely, fission is the splitting of a single mitochondrion into two or more smaller units, which allows for the removal of damaged components and for quality control. Mitochondrial fusion is regulated by dynamin-like proteins including mitofusin 1 (Mfn1), mitofusin 2 (Mfn2), and optic atrophy 1 (Opa1), whereas Dynamin-related protein 1 (Drp1), along with its receptors, i.e. mitochondrial fission factor (MFF) and fission 1 protein (FIS1), are the main regulators of mitochondrial fragmentation.

Drp1 protein, encoded by the DNM1L gene, is located in the cytoplasm and translocates to the outer mitochondrial membrane (MOM) to assemble into a spiral structure, which exerts a mechanical force on the mitochondria, leading to fission. These dynamic processes are crucial for the regulation of mitochondrial function, distribution and quality, ensuring that these organelles meet the energy demands of the cell and adapt to various stressors [6, 7]. Accordingly, balanced mitochondrial dynamics allows for proper cellular homeostasis, regulating several physiological processes such as energy production, apoptosis, and cellular responses to environmental changes [8].

Drp1 phosphorylation at S616, mediated by protein kinases as ERK2, CDK1, p38-MAPK and many others, enhances its translocation to the outer mitochondrial membrane and increases its fission-promoting activity [9]. Elevated mitochondrial fission, caused by increased expression and/or activity of Drp1, in turn drives metabolic reprogramming, cell cycle progression and cell death evasion, enhancing migration and invasiveness in a wide variety of solid cancers [10–14]. Defects in mitochondrial dynamics have been also reported in hematological malignancies, as T-cell acute lymphoblastic leukemia and MM [15, 16], which enhance the expression and/or activity of fission proteins, like Drp1, to promote survival and resistance to chemotherapy. On this basis, targeting Drp1 holds therapeutic potential in cancer [17].

Natural products currently play a relevant role in cancer therapy, with an increasing number of clinically used anti-cancer agents being either natural or derived from natural products [18]. In this regard, almost 100 natural compounds with documented anti-tumor activity have been so far studied in the context of preclinical models of MM [19–22]. Flavonoids, a class of naturally occurring compounds widely distribute in several plants, have proven useful in preventing the development of various cancer types [23, 24], triggering anti-tumor activity through still undisclosed molecular mechanisms. Herein, using MM as an investigational model, we demonstrate that two flavanones, namely Hesperetin (Hes) and Naringenin (Nar), trigger anti-cancer activity through the targeting of the mitochondrial fission pathway.

## Methods

### Molecular Docking

Molecular docking was performed on the crystallographic structure of Dynamin-1-like protein (Drp1) corresponding to PDB entry 4H1V [25]. Molecular structures of Naringenin (Nar) and Hesperetin (Hes) were built using Avogadro modeling software [26]. Docking calculations were performed by using AutoDock Vina 1.1.2 [27]. Preliminary conversion of the structures from the PDB format was carried out by the graphical interface AutoDock Tools 1.5.6 [28]; during the conversion, polar hydrogens were added to the crystallographic enzyme structures, whereas apolar hydrogens of the ligands were merged to the carbon atom they are attached to. Full flexibility was guaranteed for the ligands, resulting in four active torsions for Nar and five for Hes. As a first step, to account for the binding in any possible internal pocket of Drp1, a search volume including the whole protein was considered, with a grid spacing of 1 Å. Next, a refined search was conducted on a volume of 20 Å × 20 Å × 20 Å, considering as the centre of the search volume the position that in the crystallographic ligand is occupied by the oxygen atom of the triphosphate group bound to the tetrahydrofuran ring. A single simulation run was carried out in each case at very high exhaustiveness, 16 times larger than the default value. The binding modes of the ligands were analyzed through visual inspection, and intermolecular interactions were evaluated by using the automated protein–ligand interaction profiler, PLIP [29].

### MM cell lines and drugs

Human MM cell lines were cultured in RPMI-1640 medium supplemented with 10% of heat inactivated fetal bovine serum (FBS) (Gibco®, Life Technologies, Carlsbad, CA), 100 U/mL of Penicillin and 100 µg/mL of Streptomycin (P/S) (Gibco®, Life Technologies, Carlsbad, CA) and incubated at 37 °C in a 5% CO_2_ atmosphere. All the cell lines were periodically tested for mycoplasma contamination. NCI-H929 cell line was purchased from DSMZ (Braunschweig, Germany), which certified authentication performed by short tandem repeat DNA typing. Bortezomib (BZB) and carfilzomib (CFZ) resistant NCI-H929 cells were selected by progressive exposure to increasing concentrations of proteasome inhibitors (PIs). AMO-1 and AMO-BZB cells were kindly provided by Dr. Driessen (University of Tubingen, Germany).

Human bone marrow mononuclear cells (BMMCs) from MM patients BM aspirates were isolated by using Ficoll-hypaque (Lonza Group, Basel, Switzerland), followed by positive selection using anti-CD138 microbeads (Miltenyi Biotech, Germany), in accordance with the Declaration of Helsinki following informed consent and Institutional Review Board (University of Catanzaro, Italy) approval. Human bone marrow stromal cells (BMSCs) were obtained by long term culture of BMMCs, as previously described [30]. For co-culture experiments, MM cells were seeded on BMSCs (ratio 1:2) and cultured for 72 h in 96 well plates.

Hesperetin and Naringenin powder, as well as bortezomib and carfilzomib  DMSO solutions, were purchased from Selleck Chemicals LLC (Munich, Germany). Z-VAD-FMK (#V116) was purchased from Sigma Aldrich (USA).

### Plant material, extraction preparation and determination of aglycone flavanones

Tacle® is a citrus fruit obtained from the cross between *Citrus clementina* Hort. ex. Tanaka (Monreal Clementina) and *Citrus sinensis* L. Osbeck (tetraploid Tarocco). The samples were provided in November 2021 by “Azienda Agricola Terzeria” located in Sibari (Cosenza, Italy). Whole fresh fruits, peel and pulp were stripped of all impurities, cut and placed to macerate into a glass container for 48 h at room temperature using methanol as solvent, with a proportion of 1:10 g/mL (plant material:solvent). The procedure was repeated 3 times in order to have complete extraction of the active compounds, and the obtained solutions were combined and dried under reduced pressure using a Rotavapor® R-220 SE (BUCHI Labortechnik AG, Flawil, Switzerland). Naringenin and hesperetin quantification was determined by high-performance liquid chromatography (HPLC), as previously reported [31].

### RNA-sequencing, differential gene expression (DEG) and pathway analyses

RNA was quantified using a Qubit™ RNA HS Assay kit (Thermo Fisher Scientific) and its integrity was determined on an Agilent 2200 System by the Agilent High Sensitivity RNA Assay (Agilent). Libraries preparation was manually performed using the Ion AmpliSeq™ Transcriptome Human Gene Expression Core Panel (Thermo Fisher Scientific), according to the Ion AmpliSeq Library Kit Plus (Thermo Fisher Scientific). Briefly, 10 ng of RNA was reverse transcribed with SuperScript™ Vilo™ cDNA Synthesis Kit (Thermo Fisher Scientific). The resulting cDNA was amplified to prepare barcoded libraries using the Ion Xpress™ Barcode Adapters (Thermo Fisher Scientific), according to the manufacturer’s instructions. Barcoded libraries were combined to a final concentration of 50 pM and loaded on Ion 540™ Chips, using the Ion 540™ Kit-Chef (Thermo Fisher Scientific). Sequencing was performed on Ion GeneStudio S5™ System with Torrent Suite™ Software v5.14.0 (Thermo Fisher Scientific).

Differential gene expression (DEG) was analyzed using Transcriptome Analysis Console Software, version 4.0.3.14 (Thermo Fisher Scientific),  using threshold values for the range of fold change (< − 1.5 and >  + 1.5), p-value (< 0.05), and false discovery rate (FDR) adjusted p-value (< 0.15). Gene ontology (GO) group enrichment was performed using the ClusterProfiler package [32]. The resulting p-values were adjusted for multiple testing by the Benjamini − Hochberg procedure [33]. GO terms associated with an adjusted p-value < 0.05 were considered to be significantly enriched.

### Transmission electron microscopy (TEM)

Samples were processed according to standard protocols for ultrastructural TEM analysis. Cell pellets were fixed in 3% glutaraldehyde (Merck Sigma-Aldrich) in 0.1 M phosphate buffer at pH 7.4 (ClinicalSciences) for 2 h at 4 °C. After 2 h, three washings were carried out in phosphate buffer at 4 °C to eliminate any residual fixative. A post-fixation in 1% osmium tetroxide in phosphate buffer (0.1 M, pH 7.4) was then performed for 2 h at 4 °C to preserve the lipid structures. Samples were washed 3 times in phosphate buffer, subjected to gradual dehydration using increasing concentrations of acetone, and embedded with epoxy resin (Epon). Ultrathin sections (60–90 nm) were obtained using an RMC PowerTome series ultramicrotome with a Diatome diamond knife, collected on 300 mesh copper grids, and observed with a Jeol JEM-1400 Plus transmission electron microscope operating at 80 kV. Mitochondrial size measurements were obtained using Image J (version 1.52i, National Institutes of Health, Bethesda, MD, USA) by manually tracing mitochondria on TEM images. Statistical significance was verified using the lengths of 20 mitochondria per cell; at least 30 cells were counted for each condition. Data (presented as mean ± S.E.M.) were analyzed by the one-way analysis of variance (ANOVA) and Tukey’s test.

### Cell viability assay

Cell Titer Glo (CTG) assay kit (Promega, Madison, WI, USA) was used to evaluate cell viability, according to manufacturer’s instructions. Briefly, MM cells were seeded in 24-well plates and treated with different concentrations of Hes, Nar or Tacle extract. After 48 h, MM cells were collected, and the luminescence recorded using a GloMax-multi detection system (Promega, Madison, WI, USA). The Chou-Talalay method was employed to investigate and quantify drug combination synergism. Initially, dose–effect curves were established for bortezomib and carfilzomib, following treatment at different time points in AMO1 cell line. Subsequently, various concentrations of each proteasome inhibitor were combined with Hes or Nar (100–250 μM). Combination Index (CI) values were then calculated using the CalcuSyn software (Version 2.11; BIOSOFT, Cambridge). Drug interactions were analyzed by isobologram analysis using the CalcuSyn Version 2.0 software program (Biosoft). A combination index (CI) < 1.0 indicates synergism, CI = 1 indicates an additive effect, and CI > 1 indicates no significant combination effect [34].

For co-culture experiments, MM cell lines stably expressing the luciferase reporter gene were generated, as previously reported [35], and co-cultured with primary BMSCs (ratio 1:2) in 96 well plates. After 72 h of Hes or Nar treatment, cells were lysed, then the luciferase assay reagent added according to Luciferase assay systems protocol (Promega), and luminescence recorded using the GloMax multi-detection system (Promega). The percentage of luciferase activity was plotted after normalization to the vehicle control.

### Colony formation assay

For the colony-forming assay, MM cells were plated in triplicate in 24 well plates, in 1 mL of mixture of 1.1% methylcellulose (MethoCult STEMCELL) in RPMI-1640 supplemented with 10% FBS. The cell suspension was then allowed to grow in a humidified incubator at 37 °C for 10 days. Following incubation, colonies were fixed with methanol, stained with a crystal violet solution (0.04%), visualized and counted under a light microscope (Leica DM IL LED).

### Oxygen consumption rate (OCR) assessment via OROBOROS

Real time measurement of OCR was performed by using the high-resolution Oxygraph-2 k (O2k, Oroboros Instruments, Austria). After treatment with Hes, Nar or Tacle, MM cells were collected and loaded in the O2k-chambers at a density of 1 × 10^6^ per mL, for a total of 2 mL per chamber, allowing for parallel measurement, by maintaining cell suspension continuously mixed. Experiments were performed at a controlled temperature of 37 °C. Instrument calibration was performed daily. First, routine respiration was measured in basal cellular conditions (before adding reagents), then variations of respiration were evaluated after sequential addition of Oligomycin A (2 µM), FCCP (0.5 µM), and Antimycin A (2 µM). Oroboros reagents, including Oligomycin (#O4876), FCCP (#C2920) and Antimycin A (#A8674), were purchased from Sigma Aldrich. Reagents were added into the closed chamber by Hamilton syringes (Oroboros Instruments, Innsbruck, Austria). Measurements were normalized for the cell count. Traces were analysed by DatLab 7 software. O_2_ flow is expressed per cell [amol·s^−1^·x^−1^] equivalent to [pmol·s^−1^·(10^6 ^cells)^−1^].

### Apoptosis detection

Apoptosis was evaluated by Annexin V/7-Aminoactinomycin D (7-AAD) flow cytometry assay (BD biosciences). MM cells were stained in a 5 mL polystyrene tube, following the protocol of the PE/Annexin V Apoptosis Detection Kit (Thermo Fisher Scientific, Waltham, MA, USA). After Hes and Nar treatments, MM cells were collected, washed with PBS 1X, and exposed to Annexin V-PE and 7-AAD probes for 15 min at room temperature. Samples were analysed using a FACS Fortessa X-20 instrument (BD Biosciences, San Jose, CA, USA); data were analyzed using the FlowJo software version 10 and presented as histogram bars, indicating the percentage of early and late apoptotic cells.

### Western blotting (WB)

Total protein extracts were prepared using NP40 Cell Lysis Buffer supplemented with Halt Protease Inhibitor Single-Use Cocktail (Thermo Fisher Scientific, Waltham, MA, USA). Protein samples (30 μg) were run in 10% SDS-PAGE and then transferred to nitrocellulose membranes using the Trans-Blot Turbo Transfer System (Bio-Rad Laboratories, Hercules, CA, USA); membranes were next subjected to immunoblotting using the following primary antibodies: Drp1 (#14647S), p-Drp1 S616 (#3455S), Ubiquitin (#3933S), ATF4 (#11815S), PERK (#5683), p-EIF2α (#3398), Caspase 3 (#9665P), PARP (#9532), c-MYC (#2276S), FAS (#3180 T), ACC (#3676 T), ACL (#4332 T), α-TUBULIN (#2125S), and GAPDH (#5174), all from Cell Signaling Technologies; SREBP1 antibody (sc-365513) was from Santa Cruz Biotechnology (Dallas, TX, USA); DGAT2 antibody (PA5103785) was from Thermo Fisher Scientific.

### Lentivirus production and transduction of MM Cells

MM cells stably expressing the luciferase transgene or DNM1L gene were obtained as previously described [21]. The packaging of each construct in pseudoviral particles was performed in HEK-293Ta cells, which were co-transfected with 10 µg of the lentiviral Lenti-hCMV-ORF-N-HA-IRES-puro for Homo sapiens DNM1L (or the empty vector, both from Transomic), along with 10.0 µg of p-CMV-VSVG, and 4.0 µg of Delta8.9 plasmids. After 48 h, supernatants containing lentiviral particles were collected and filtered through 0.45 µm filters, then 2 mL were used for a single round of overnight transduction of MM cells (0.5 × 10^6^), in the presence of 8 µg/ml polybrene (Sigma Aldrich, St. Louis, MO). Two days after transduction, MM cells were selected in medium containing 0.5 µg/ml puromycin for three weeks.

### Triglyceride assay

Triglyceride-Glo assay (Promega, Madison, WI, USA) was used to evaluate total triglyceride levels, according to manufacturer’s instructions. MM cells were seeded in 24-well plates and treated with 250 µM of either Hes or Nar, or with 2 mg/ml Tacle extract. After 48 h, MM cells were collected, and luminescence recorded using a GloMax-multi detection system (Promega, Madison, WI, USA).

### Reverse transcription and quantitative real-time amplification (qRT-PCR)

RNA extraction, reverse transcription (RT), and quantitative real-time amplification (qRT-PCR) were carried out following previously described methods [36]. Total RNA was extracted from MM cells using TRIzol Reagent (Thermo Fisher Scientific, Waltham, MA, USA) according to the manufacturer's protocol; the integrity and purity of the isolated total RNA were assessed using a Nanodrop Spectrophotometer nd-2000 (Thermo Fisher Scientific, Waltham, MA, USA). To quantify the expression levels of SREBF1 (Hs01088679_g1; Applied Biosystems) and c-MYC (Hs01570247_g1; Applied Biosystems) genes, a single-tube TaqMan mRNA assay (Applied Biosystems, Thermo Fisher Scientific, USA) was employed, following manufacturer's instructions. To quantify the expression levels of ATF4, PERK and sXBP1, a Syber Green assay provided by Applied Biosystems was employed following manufacturer's instructions, using the QuantStudio 12 K Flex reader (Thermo Fisher Scientific, Waltham, MA, USA). The sequence of primers, purchased from Eurofins, was the following: ATF4 forward (5ʹ-TTCTCCAGCGACAAGGCTAAGG-3ʹ), ATF4 reverse (5ʹ-CTCCAACATCCAATCTGTCCCG-3ʹ), PERK forward (5ʹ-GTCCCAAGGCTTTGGAATCTGTC-3ʹ), PERK reverse (5ʹ-ATCCTACCAAGACAGGAGTTCTGG-3ʹ), sXBP1 forward (5ʹ-AGACAGCGCTTGGGGATGGAT-3ʹ), sXBP1 reverse (5ʹ-CCTGCACCTGCTGCGGACTC-5ʹ), β-Actin forward (5ʹ-CAAGGCCAACCGCGAGAAGATGAC-3ʹ), β-Actin reverse (5ʹ-GCCAGAGGCGTACAGGGATAGCACA-3ʹ). Relative expression was calculated using the comparative threshold (Ct) method.

### Immunofluorescence staining of lipid droplets

Lipid droplets were determined using LipidTOX (Thermo Fisher Scientific) or BODIPY 493/503 (Thermo Fisher Scientific) probe staining, according to standard protocols. Briefly, after Hes and Nar treatments, MM cells were fixed with 4% paraformaldehyde for 15 min, followed by permeabilization with 0.2% Triton X-100 for 10 min. Cells were then incubated in the dark with HCS LipidTOX™ Red Neutral Lipid Stain (cat. H34476) or BODIPY 493/503 (cat. D3922) for 30 min and 20 min, respectively. Finally, after two washes with 1X PBS, a mounting medium containing DAPI, for nuclear staining, was added to cells. Images were obtained under a Leica DM4 B fluorescence microscope. Lipid staining intensity was quantified by measuring the mean fluorescence intensity using the ImageJ software.

### In vivo studies

Male SCID/NOD mice (6- to 8-weeks old; Envigo, Indianapolis) were housed and monitored in our Animal Research Facility. All the experimental procedures and protocols were previously approved by the Institutional Ethical Committee (Magna Graecia University) and performed according to protocols approved by the National Directorate of Veterinary Services (Italy). According to institutional guidelines, mice were sacrificed when tumors reached 2 cm in diameter or in the case of paralysis or significant compromise in their quality of life, in order to mitigate unnecessary suffering. Mice were subcutaneously inoculated with 5.0 × 10^6^ AMO1 cell line; when tumors became palpable (100–200 mm^3^; ≈3 weeks after injection), mice were divided into two groups (n = 5/group), and intraperitoneally (i.p.) treated with Tacle extract (25 mg/kg) or vehicle as control, 5 days a week for a total of two weeks. Tumor volume was measured by an electronic Caliper using the following formula: *V* = *(a*^*2*^* x b)/2*; where *a* is the width and *b* is the length of the tumor.

### Immunohistochemistry (IHC)

Retrieved tumors and organs from treated mice were immersed in 4% buffered formaldehyde, and 24 h later washed, dehydrated, and embedded in paraffin. Hematoxylin–eosin (H&E) staining was performed on 4 μm sections mounted on polylysine slides. Tissues sections were observed under the OLYMPUS BX51optical microscope (Olympus Corporation, Tokyo, Japan). For IHC staining, tumor and organs slices (2 μm size) were deparaffinized and pre-treated with the Epitope Retrieval Solution 2 (EDTA-buffer, pH 8.8) at 98 °C for 20 min. After washing steps, peroxidase blocking was carried out for 10 min using the Bond Polymer. All procedures were performed using the Benchmark XT-Automated Immunohistochemistry instrument (Ventana Medical Systems, Oro Valley, AZ, USA). Tissues were again washed and then incubated with the primary antibody directed against Ki67 (Dako, clone: MIB-1; 1:150). Subsequently, tissues were incubated with polymer for 10 min and developed with DAB-Chromogen for 10 min. The experiments were repeated at least three times.

### Statistical analysis

In vitro experiments were performed at least three times and reported as mean ± SD. Comparison between groups was made using Student’s t-test, whereas statistical significance of differences among multiple groups was determined by GraphPad software (www.graphpad.com). Graphs were obtained by GraphPad Prism software (version 8; La Jolla, CA, USA). A p-value < 0.05 was accepted as statistically significant.

## Results

### Hesperetin and Naringenin target mitochondrial fission in MM cells

We previously reported a high content of flavonoids in extracts of Tacle^®^, a hybrid citrus plant obtained from the cross-breeding of Clementine and Tarocco tetraploids, largely farmed in the Sibari plane (Southern Italy). In these extracts, the most abundant components are represented by two flavanones, namely Hesperedin and Naringin, known for their anti-oxidant and lipid-lowering activity. Since the majority of the flavonoid glycosides are hydrolysed at intestinal or hepatic level, the free corresponding aglycones Hesperetin (Hes) and Naringenin (Nar), which reach high concentrations in the systemic bloodstream, seem to account for the biological activity [37, 38].

HPLC analysis indicated that flavanones were the most abundant compounds in Tacle extract (Additional file 1: Fig. S1).

Recent studies have shown the potential of some flavonoids in modulating mitochondrial functions [39]. To verify whether Hes and Nar could interact with Drp1, thus affecting mitochondrial dynamics, molecular docking studies were performed on the crystallographic structure of the catalytic portions of the protein retrieved from the Protein Data Bank (PDB code: 4H1V). In this structure, the target protein is complexed with the nucleotidic ligand GNP (phosphoaminophosphonic acid-guanylate ester).

In the first step of the simulation procedure, re-docking experiments were performed to calculate the binding energy value for the crystallographic ligand GNP into Drp1 binding site. As a result, a -8.1 kcal/mol value was obtained and taken as a reference. Next, Hes and Nar were docked into Drp1, and both compounds were found to accommodate in the binding site of the protein with favourable binding energy values (− 7.6 and − 7.4 kcal/mol for Nar and Hes, respectively), interacting with key amino acid residues of the active site (Additional file 1: Fig. S2).

**Fig. 1:**
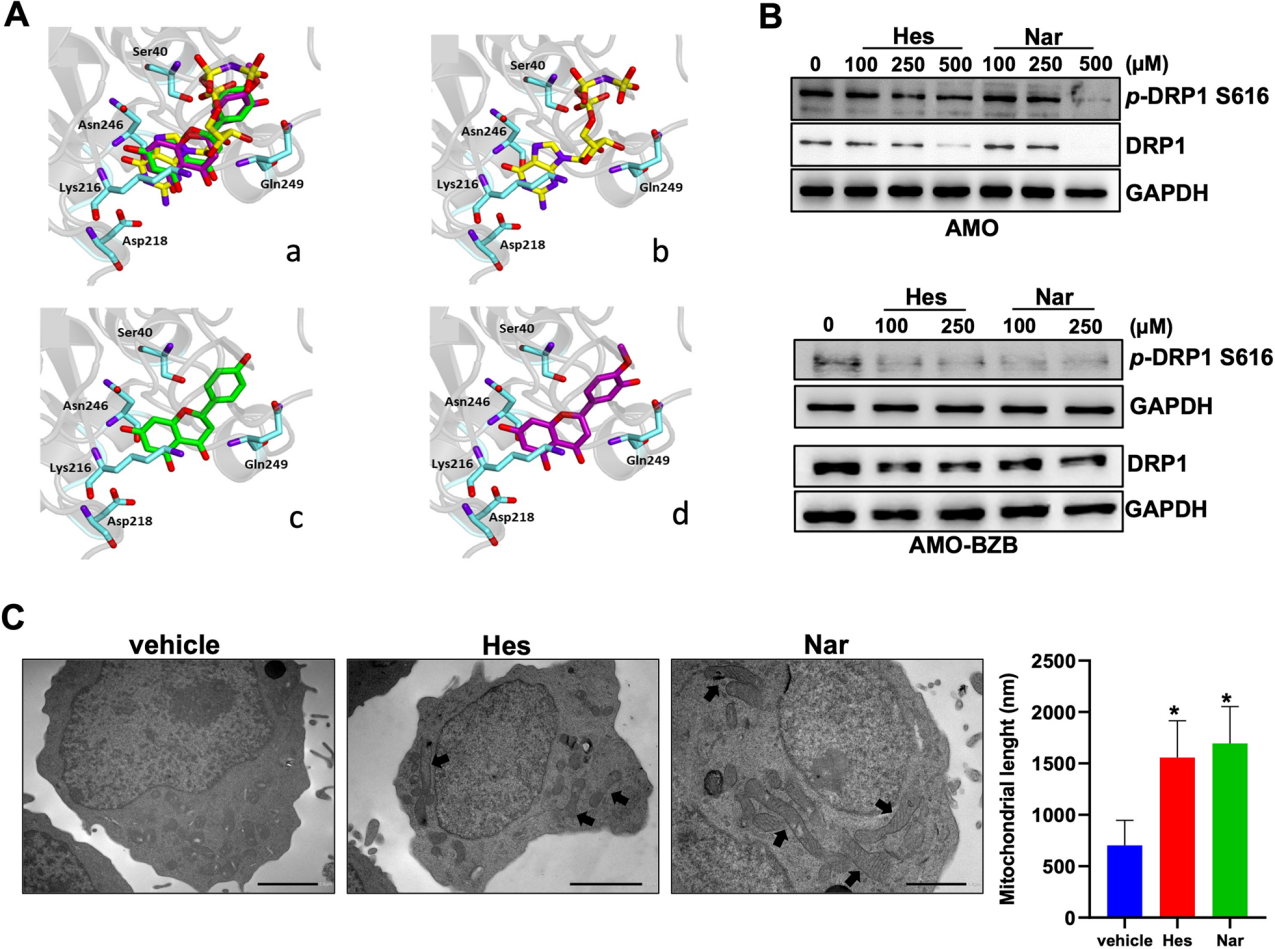
Hes and Nar selectively inhibit Drp1. **A** Ligand-binding pocket of the active site of Drp1. Protein backbone is represented in background as a ribbon, and key protein residues are in blue. (a) Superimposed binding modes of the crystallographic ligand GNP (yellow); Nar (green) and Hes (magenta). The ligands are also shown separately: (b) crystallographic ligand GNP (yellow), (c) NAR and (d) HES. **B** WB analysis of Drp1 and S616 p-DRP1 was performed in AMO and AMO-BZB cells, 48 h after treatment with increasing doses of Hes, Nar or DMSO as vehicle. GAPDH was used as loading control. **C** TEM analysis (12000x) of mitochondrial structure and morphology in AMO cell line, 24 h after exposure to 100 µM of Hes or Nar. Representative images are shown; arrows indicate elongated mitochondria

Overall, Hes and Nar displayed a similar orientation within the active site (Fig. 1A), interacting through hydrogen bonds with Ser40, Lys216, Asp218, Asn246 and Gln249, all residues also involved in the interaction of Drp1 with a synthetic ligand [40]; the complexes were further stabilized by hydrophobic interactions with Lys216 and Asn246 residues.

Consistent with an inhibitory role on mitochondrial fission, WB analysis showed that Hes and Nar treatment of AMO or AMO-BZB cells reduced the levels of S616 phosphorylated-Drp1 and total Drp1 (Fig. 1B), and increased the number of fused mitochondria, as determined by TEM (Fig. 1C); conversely, the levels of the mitochondrial fusion protein Mfn2 did not change (Additional file 1: Fig. S3). Overall, these data suggest that Hes and Nar are promising natural candidate molecules targeting the mitochondrial fission executioner Drp1.

### Hesperetin and Naringenin suppress OXPHOS reducing MM cell viability

**Fig. 2:**
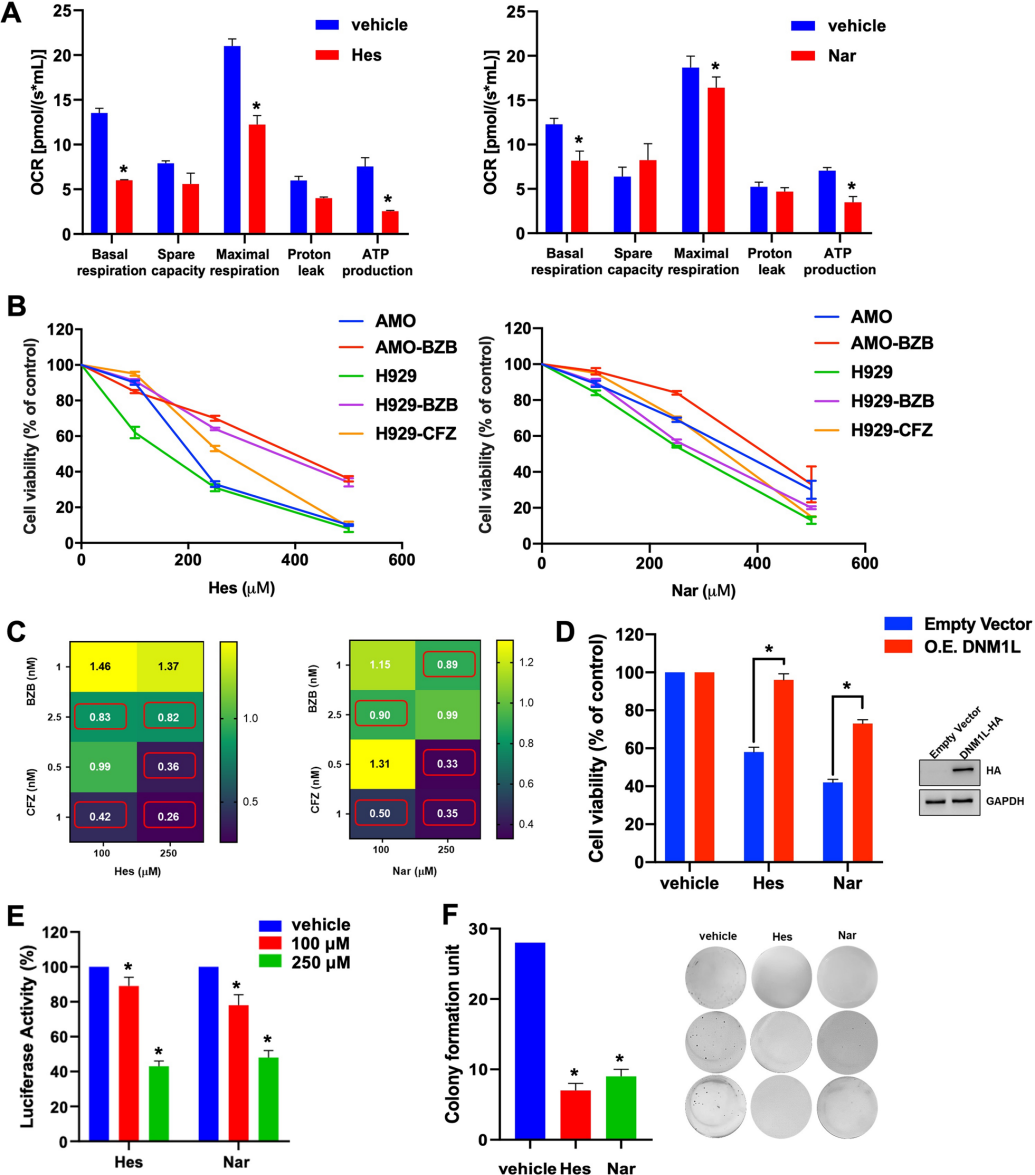
Hes and Nar affect the viability of MM cell lines. **A** Real time Oxygen Consumption Rate (OCR) measurement in closed chambers performed on H929 cell line, 48 h after Hes (250 µM—*left panel*) or Nar (250 µM—*right panel*) treatment. Histogram bars report multiple key parameters, including basal respiration, spare capacity, maximal respiration, leak state and ATP production, calculated following consecutive injections of 2 µM Oligomycin, 0.5 µM FCCP and 2 µM Antimycin A. *p < 0.05. **B** Cell viability was assessed by CTG assay, 48 h after treatment with increasing doses of Hes (*left panel*) or Nar (*right panel*), as compared to vehicle (DMSO). Viable cells are reported as percentage of DMSO-treated cells. *p < 0.05. **C** Heat-map showing combination indexes (CI), determined by Calcusyn software, in AMO cells, 48 h after combined treatment of Hes (100–250 µM; *left panel*) or Nar (100–250 µM; *right panel*), with BZB (1–2.5 nM) or CFZ (0.5–1 nM). **D** Cell viability assessed by CTG assay in AMO cells stably overexpressing DNM1L gene, 72 h after 100 µM Hes or 100 µM Nar exposure. Immunoblot shows protein levels of HA-tagged Drp1 after transduction; GAPDH was used as loading control. *p < 0.05. **E** Cell viability assessed by Luciferase Glo assay in luciferase-expressing AMO cells, co-cultured in adhesion to BMSCs and exposed to Hes and Nar for 48 h. Cell viability was expressed as percentage of luciferase activity with respect to DMSO-treated cells. Data represent the average ± SD of three independent experiments. *p < 0.05. **F** Colony formation assay performed on AMO cells exposed to 250 µM Hes or 250 µM Nar for 10 days; DMSO was used as vehicle. Histogram bars represent the mean ± SD of three independent experiments. Representative images of colonies at day 10 are reported

Previous studies indicate that, when mitochondrial fission is dampened, tumor cells change their mitochondrial respiration rewiring their metabolic activities [41]. Worthy of note, high resolution respirometry in AMO cells evidenced a reduction of basal oxygen consumption rate (OCR), along with a decrease in maximal respiratory capacity, and ATP production, upon Hes or Nar treatment as compared to vehicle (Fig. 2A; Additional file 1: Fig. S4). To evaluate the impact of the two flavanones on MM cell viability, we performed a CTG assay, using MM cells sensitive or resistant to proteasome inhibitors (PIs), one of the most relevant therapeutic option in MM [42]. Our results indicate that both Hes and Nar significantly inhibited the growth of PI-sensitive (AMO and NCI-H929) or resistant (AMO-BZB, H929 BZB, H929-CFZ) MM cell lines in a dose-dependent manner (Fig. 2B; IC_50_ values are reported in Additional file 1: Fig. S5); as previously reported, no cytotoxic effect was observed on healthy PBMCs (data not shown) [43], suggesting a favorable therapeutic window. Moreover, as determined by Calcusyn analysis, Hes and Nar synergistically enhanced the activity of the PIs bortezomib (BZB) and carfilzomib (CFZ; Fig. 2C).

Importantly, lentiviral-mediated overexpression of DNM1L gene, encoding Drp1 protein, almost completely abrogated Hes and Nar effects on MM cell viability (Fig. 2D), indicating that their anti-MM activity mainly occur in a Drp1-dependent fashion. We also investigated the effect of Hes and Nar on MM cells co-cultured with bone marrow stromal cells (BMSCs), which are known to promote MM cell survival and drug resistance [44]. Notably, Hes and Nar treatment antagonized the protective effect of BMSCs on MM cells (Fig. 2E), suggesting that these compounds have the potential to disrupt the microenvironmental interactions prompting MM cell survival. Additionally, treatment with Hes or Nar significantly decreased the colony formation ability of MM cells (Fig. 2F), strengthening the anti-tumor potential of these two compounds.

### Hesperetin and Naringenin trigger the ER stress-apoptotic response in MM cells

**Fig. 3:**
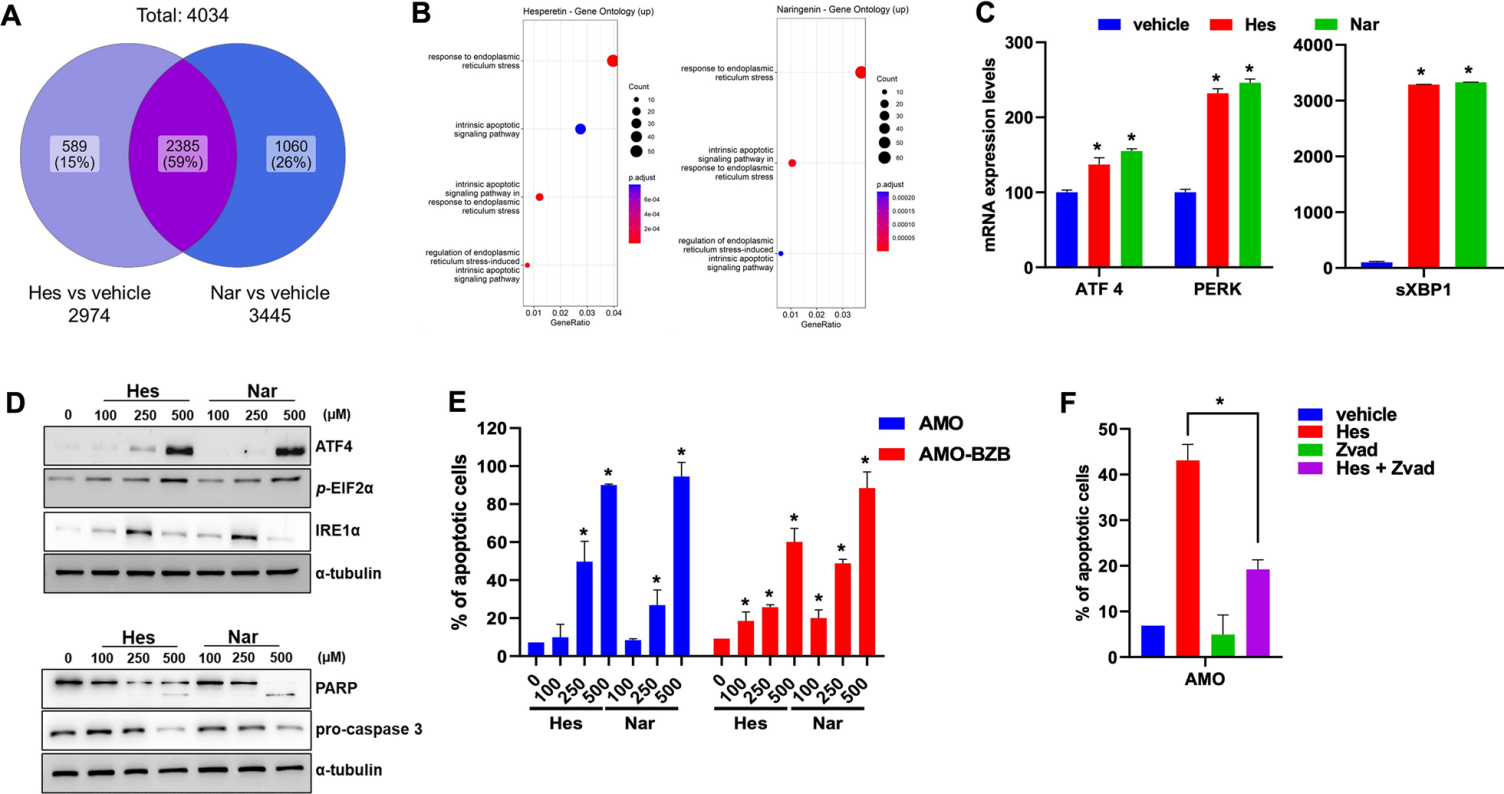
Hes and Nar activate ER-stress and apoptosis.** A** Venn diagram reporting the differentially expressed genes induced by the two treatments and their comparison. **B** The plot shows the Gene Ontology (GO) enrichment of the upregulated genes after Hes and Nar treatment. The size of the dot indicates the number of upregulated genes enriched in each pathway, while the color of the dot indicates the p-adjusted value. **C** q-RT-PCR analysis of ATF4, PERK and sXBP1 mRNAs, in AMO cells, 24 h after treatment with 250 µM of either Hes or Nar. Histogram bars represent the average ± SD of mRNA expression levels after normalization with GAPDH and ΔΔCt calculations. **D** WB analysis of ATF4, IRE1α, p-EIF2α, PARP and Caspase 3 (*right panel*) proteins in AMO cells, 48 h after Hes or Nar treatment; normalization was performed using GAPDH or α-tubulin as loading controls. **E** FACS analysis of Annexin V-positive AMO and AMO-BZB cells, 48 h after Hes or Nar exposure. Data are representative of at least three independent biological replicates (n = 3). Histogram bars reported the percentage of total apoptotic cells. *p < 0.05. **F** FACS analysis of Annexin V-positive cells, 48 h after treatment with Hes (250 µM) alone or in combination with Z-VAD (20 µM). Data are representative of at least three independent biological replicates (n = 3). Histogram bars report the percentage of apoptotic MM cells. *p < 0.05

To decipher the molecular perturbations underlying Hes and Nar activity in MM cells, we performed gene expression profiling analysis using the Ion AmpliSeq platform. The Venn diagram in Fig. 3A provides an overview of the DEG analysis: a total of 4034 genes were differentially expressed after Hes and Nar treatments. Specifically, 2974 transcripts were differentially modulated in Hes treatment versus vehicle, whereas 3445 were differentially expressed in Nar treatment compared to vehicle cells. Moreover, 589 and 1060 DEGs were evident only after Hes or Nar single treatment, respectively; conversely, 2385 DEGs were common in both treatments, with respect to control. Next, we performed Gene Ontology enrichment analysis to evaluate the effect of Hes or Nar treatment in terms of cellular localization, molecular function, and biological processes. Interestingly, we found that the most significantly (p < 0.05) hyperactivated categories included the endoplasmic reticulum (ER) stress-induced response and apoptosis for both Hes and Nar (Fig. 3B). Consistently, qRT-PCR analysis evidenced the upregulation of ATF4, PERK and spliced XBP1 (sXBP1) (Fig. 3C), and WB confirmed the increased phosphorylation of eIF2a and the induction of ER stress sensor proteins, namely IRE1α and PERK, and of ATF4, as well as the presence of apoptotic markers, including cleaved caspase 3 and PARP1 (Fig. 3D). Moreover, Hes and Nar increased the number of Annexin-V positive cells (Fig. 3E; Additional file 1: Fig. S6), and this effect was almost abrogated by the pan-caspase inhibitor ZVAD-fmk (Fig. 3F), further confirming apoptosis induction upon treatment with the two flavanones. Overall, these findings indicate that Hes and Nar anti-MM effects involve the activation of ER-stress and apoptosis.

### Hesperetin and Naringenin rewire MM cell metabolism through lipogenesis impairment

**Fig. 4:**
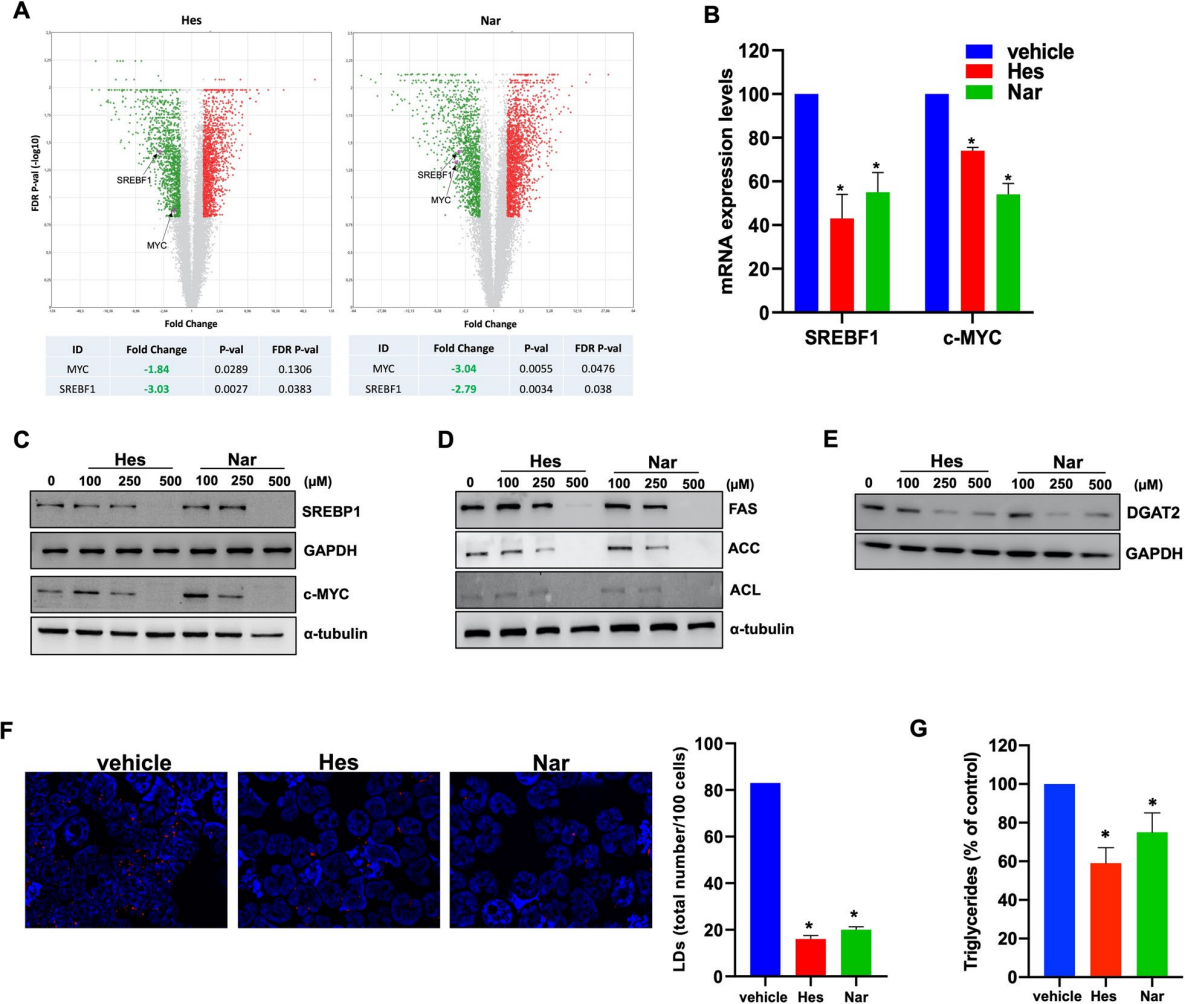
Hes and Nar target the lipogenesis pathway. **A** Volcano plot representation of differentially expressed genes after Hes (*left panel*) and Nar (*right panel*) treatment. The x-axis indicates the expression fold change (FC) and the y-axis indicates the false discovery rate (FDR) (− log_10_) for each gene versus untreated MM cells. FC threshold |1,5|; FDR ≤ 0.15. Upregulated transcripts (red) and downregulated (green) are shown. Selected representative genes are indicated with black arrows. **B** q-RT-PCR analysis of SREBF1 and c-MYC mRNA expression levels in AMO cells, 48 h after 250 µM Hes or Nar exposure. Histogram bars represent the average ± SD of mRNA expression levels after normalization with GAPDH and calculation using ΔΔCt method. *p < 0.05. WB analysis of **C** SREBP1, c-MYC **D** FAS, ACC, ACL and **E** DGAT2 protein levels, in AMO cells, 48 h after treatment with Hes or Nar. GAPDH or α-tubulin were used as loading control. **F** Fluorescence microscopy analysis of lipid droplets labeled with LipidTOX Red probe, 24 h after exposure to 250 µM of Hes or Nar. Representative images (100 × magnification) are reported. Histogram bars report the number of lipid droplets ± SD in at least 100 cells from three different fields. *p < 0.05. **G** Triglycerides measurement using Triglycerides Glo assay in AMO cells after 250 µM Hes or 250 µM Nar treatment. Luminescence was evaluated at 48 h. *p < 0.05

Data from our group and others have shown the lipid-lowering effects of citrus flavonoids [31, 45]. Aberrant mitochondrial fission in cancer has been reported to rewire lipid metabolism by promoting the expression of master transcriptional activators of lipogenesis genes [46]. The volcano plots illustrated in Fig. 4A identify the transcripts differentially expressed in MM cells challenged with Hes or Nar alone, compared to vehicle. Among those commonly down-regulated by Hes and Nar, we identified SREBF1 and c-MYC, encoding for transcription factors promoting the expression of lipogenesis genes [47]. This analysis was validated by qRT-PCR and WB, which confirmed a down-regulation of SREBF1 and c-MYC both at mRNA (Fig. 4B) and protein (Fig. 4C) level, resulting in reduced expression of lipogenesis-related enzymes, including fatty acid synthase (FAS), acetyl-Coa carboxylase (ACC), and ATP-citrate lyase (ACL) (Fig. 4D). Consistently, a decrease in DGAT2, the rate-limiting enzyme catalyzing the terminal step in the synthesis of triglycerides in lipid droplets [48] (Fig. 4E), as well as in lipid droplets (Fig. 4F; Additional file 1: Fig. S7) and triglyceride levels (Fig. 4G), could be observed after Hes or Nar treatment, suggesting that both flavanones can interfere with lipid synthesis and storage in MM cells. Overall, these observations indicate that Hes and Nar can antagonize a transcriptional network triggering lipogenesis in MM cells.

### Tacle phenocopies Hes and Nar effects triggering in vitro and in vivo anti-MM activity

**Fig. 5:**
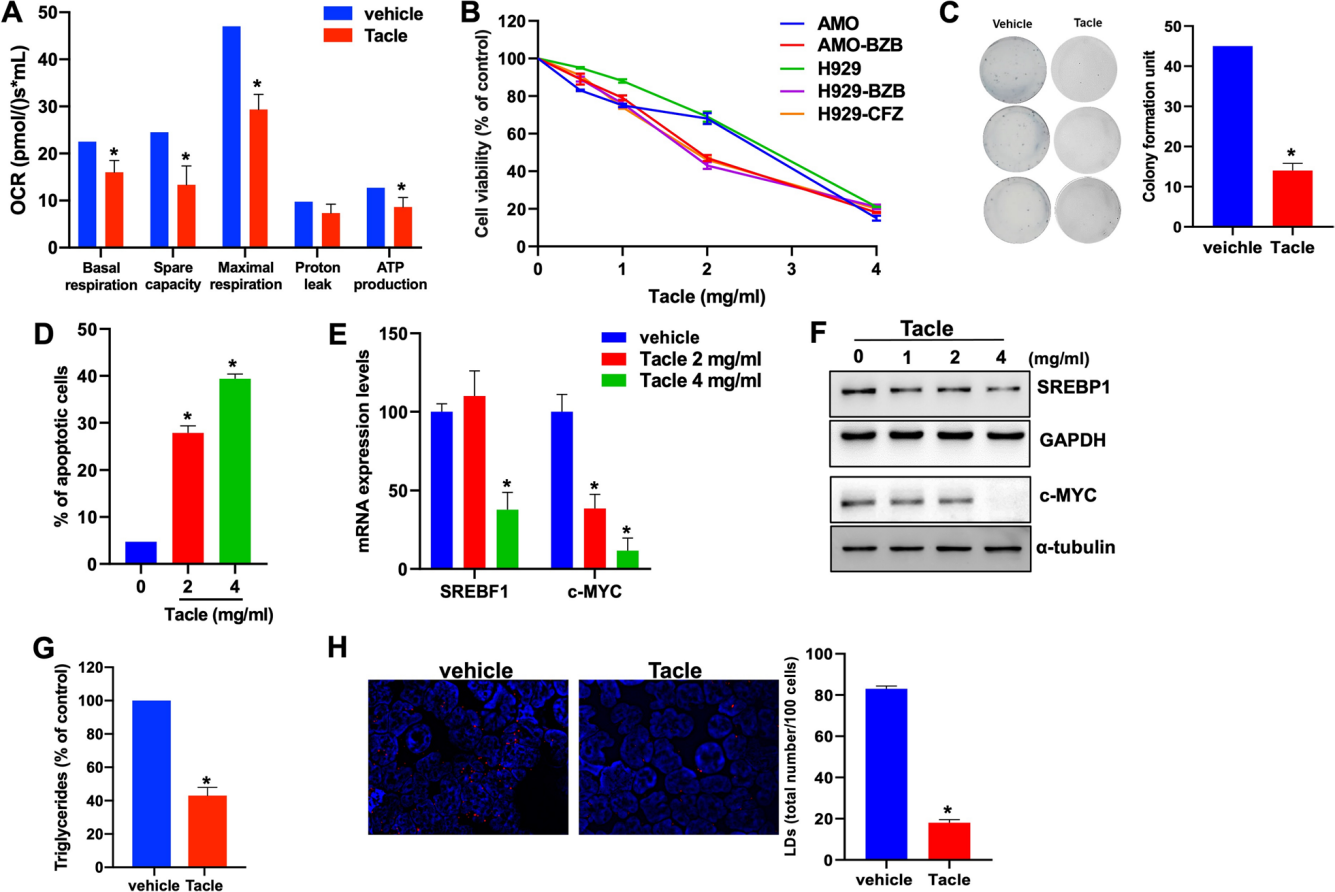
Tacle extract recapitulates Hes and Nar effects in vitro.** A** Real time OCR measurement in closed chambers performed in H929 cells, 48 h after 2 mg/mL Tacle treatment. Histogram bars report multiple key parameters, including basal respiration, spare capacity, maximal respiration, leak state and ATP production, calculated after consecutive injections of Oligomycin (2 µM), FCCP (0.5 µM) and Antimycin A (2 µM). **B** Cell viability assessed by CTG assay, 48 h after treatment with increasing doses of Tacle extract or vehicle. Live cells are represented as percentage of vehicle-treated cells. *p < 0.05. **C** Colony formation assay performed in AMO cells exposed for 10 days to Tacle extract (2 mg/mL), or vehicle as control. Histogram bars reported the mean ± SD of colony formation units of three independent experiments. Representative images of colonies at day 10 are also shown. *p < 0.05. **D** Flow cytometry evaluation of Annexin V-positive AMO cells, after 48 h of Tacle extract treatment. Data are representative of at least three independent biological replicates (n = 3). *p < 0.05. **E** q-RT-PCR analysis of SREBF1 and c-MYC mRNA expression levels in AMO cells, 48 h after Tacle exposure. Histogram bars represent the average ± SD of mRNA expression levels after normalization with GAPDH and ΔΔCt calculation method. *p < 0.05. **F** WB analysis of SREBP1 and c-MYC in AMO cells, 48 h after Tacle treatment. GAPDH or α-tubulin were used as loading control. *p < 0.05. **G** Triglycerides levels were determined using Triglycerides Glo assay in AMO cells after 2 mg/ml Tacle treatment; luminescence was evaluated at 48 h. *p < 0.05. **H** Fluorescence microscopy analysis of lipid droplets in AMO cells labeled with LipidTOX Red probe, 24 h after exposure to Tacle (2 mg/ml); representative images (100 × magnification) are reported. Histogram bars represented the average ± SD of the number of lipid droplets in at least 100 cells from three different fields

Since the extract of Tacle, a hybrid citrus variety combining Tarocco and Clementina fruits, is rich in Hes and Nar [45], we evaluated whether it could reproduce Hes and Nar effects at the phenotypic and molecular level. In line with the above-reported results, Tacle triggered a reduction of basal OCR, maximal respiratory capacity and ATP production (Fig. 5A; Additional file 1: Fig. S8); moreover, a reduction in cell viability of both PI-sensitive and resistant MM cell lines (Fig. 5B), as well as a decrease in colony formation (Fig. 5C), could be noticed after Tacle exposure. Similarly to Hes and Nar, Tacle extract decreased S616-phosphorylated and total Drp1 (Additional file 1 Fig. S9A). Additionally, activation of ER stress, leading to apoptosis, could be detected upon Tacle extract treatment, as indicated by the increase in Annexin V-positive cells (Fig. 5D; Additional file 1: Fig. S9B, C).

At the molecular level, Tacle treatment resulted in a reduction of SREBF1 and c-MYC levels (Fig. 5E, F), which was accompanied by a decrease in triglycerides (Fig. 5G), in DGAT2 (Additional file 1: Fig. S9D) and lipid droplet content (Fig. 5H; Additional file 1: Fig. S9E). DNM1L-lentiviral overexpression significantly antagonized the inhibition in triglycerides levels and lipid droplets elicited by Hes, Nar or Tacle, indicating that the lipid-lowering effects triggered by these agents in MM cells occur in a Drp1-dependent manner (Additional file 1: Fig. S10).

**Fig. 6:**
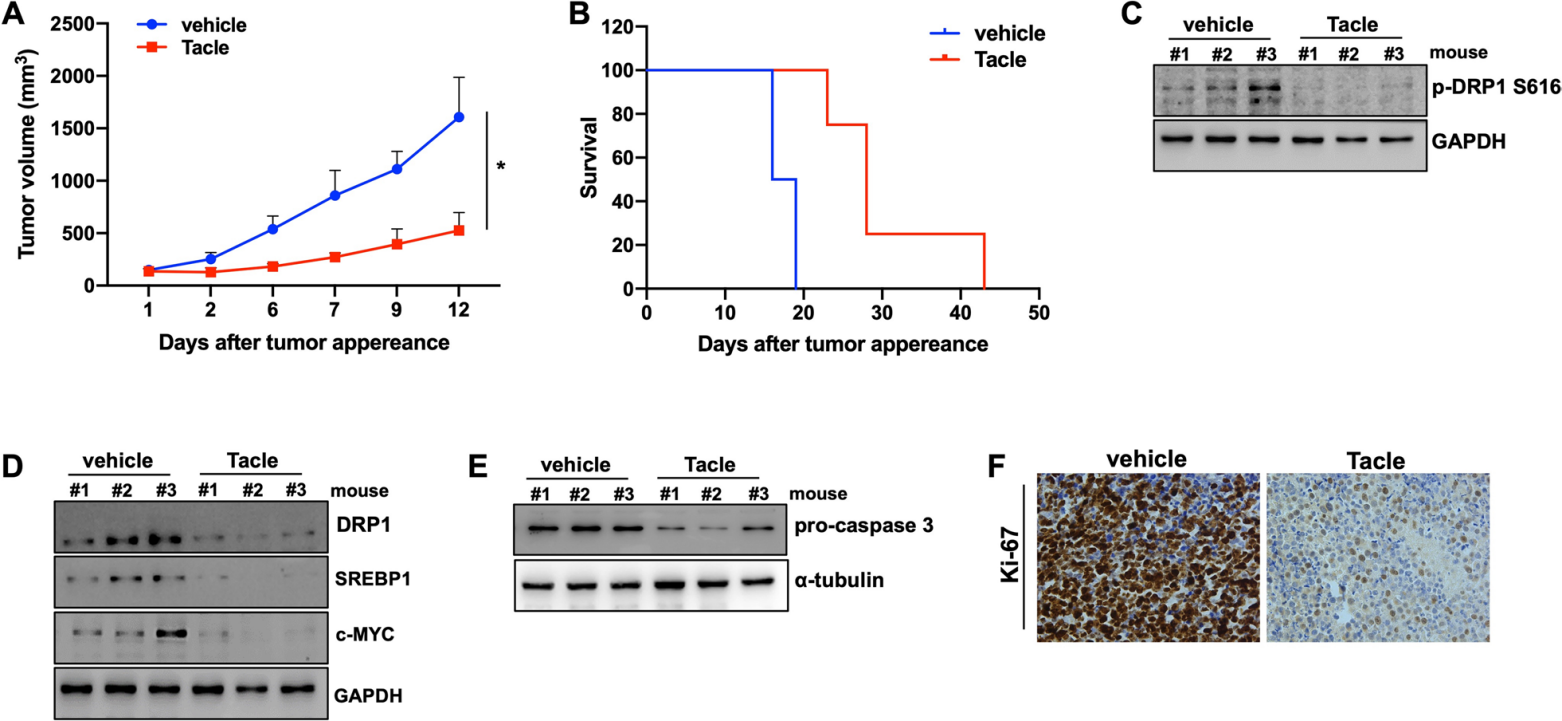
Tacle extract exerts anti-MM activity in vivo. **A** In vivo tumor growth evaluation of subcutaneous AMO xenografts receiving i.p. Tacle (250 mg/kg), or vehicle as control; administrations were performed 5 days a week for a total of two weeks. Average ± SD of the tumor volume for each group is shown; *p*-values were obtained using two-tailed t-test. *p < 0.05. **B** Kaplan–Meier curves relative to i.p. Tacle-treated AMO xenografts compared to control xenografts (log-rank test, *p < 0.05). Survival was evaluated from the first day of treatment until death or sacrifice. Percentage of mice alive is shown. WB analysis of **C** S616 p-DRP1, **D** Drp1, SREBP1, c-MYC and **E** pro-Caspase 3 protein levels in tumors retrieved from Tacle- or vehicle-treated mice. GAPDH was used as loading control. **F** IHC analysis (20 × magnification) of Ki-67 expression in AMO xenografts retrieved from mice, two weeks after Tacle treatment; representative images are reported

Finally, we performed a proof-of-concept study of Tacle anti-MM activity in vivo. To this aim, we evaluated the effects of Tacle on NOD-SCID mice-bearing NCI-H929 xenografts. I.p. administration of Tacle, five days/week for two consecutive weeks, resulted in a significative reduction in tumor volume (Fig. 6A), while prolonging animal survival as compared to vehicle-treated control mice (Fig. 6B). According to in vitro data, WB analysis of retrieved xenografts showed downregulation of total and S616-phosphorylated Drp1 (Fig. 6C), as well as reduced expression of c-MYC, SREBP1 (Fig. 6D) and of pro-caspase 3 (Fig. 6E) after Tacle extract administration, confirming in vivo the targeting of the mitochondrial fission pathway, along with the inhibition of lipogenesis and the induction of apoptosis. Consistent with an inhibitory effect on MM growth, IHC analysis evidenced a decrease in Ki67 staining in Tacle-treated retrieved xenografts (Fig. 6F). Importantly, H&E staining did not reveal any architectural change in the retrieved vital organs (heart, spleen, liver and kidneys), suggesting that Tacle extract is safe and does not induce relevant sign of toxicity in mice (Additional file 1: Fig. S11); lack of toxicity was also supported by the absence of neurological changes or weight loss of treated animals (data not shown).

## Discussion

An increasing number of studies has highlighted cancer-associated defects in mitochondria, which can be harnessed for novel personalized treatments [8]. Mitochondrial alterations prompt metabolic and phenotypic changes of tumor cells, which have been also implicated in the onset and progression of MM. MM plasma cells rely on glucose metabolism by enhancing glucose uptake, aerobic glycolysis and producing lactate, activating either the tricarboxylic acid cycle and OXPHOS, necessary to maintain elevated protein synthesis, folding and secretion, or the pentose phosphate pathway to boost the generation of the antioxidant molecules [49]. Importantly, metabolic reprogramming of MM cells is also modulated by BMSCs, which surround and protect malignant PCs producing pro-survival growth factors and cytokines; in turn, MM cells induce BMSCs to undergo metabolic changes, producing metabolites for their utilization by neoplastic cells, as OXPHOS substrates, thus sustaining ATP production and increasing cell fitness to foster growth and migration [50, 51]. Finally, the reciprocal transfer of mitochondria between MM cells and BMSCs can contribute to sustain oxidative stress associated to proteostasis [50, 52].

The maintenance of a healthy mitochondrial network, fundamental to support cell energy demand, is strictly dependent on mitochondrial fission and fusion dynamics, a process regulating changes in mitochondrial size and shape, growth and redistribution, as well as intervening in mitochondrial cristae remodeling [53]. Such a “mitochondrial quality control” system has been found frequently deregulated in pathological conditions, including cardiovascular diseases [8, 54], non-alcoholic fatty liver disease [55], acute kidney injury [56], neurodegenerative disorders [57, 58], diabetes mellitus [59], skeletal muscle atrophy [60], and many others.

Tumors can also derange the mitochondrial dynamics rheostat to gain proliferative and survival advantages. Accordingly, Drp1-dependent increased mitochondrial fission has been reported in several solid [10–14] and haematological malignancies, including MM [15, 16], overall contributing to metabolic reprogramming, cell death evasion, migration and invasiveness of tumor cells. On this basis, the selective antagonism of mitochondrial fission via Drp1 targeting might represent a promising therapeutic approach for cancer, and Drp1 synthetic inhibitors have proven effective in reducing proliferation and inducing apoptosis in cancer cells, both in vitro and in vivo [61, 62]. However, the repertoire of currently available Drp1 inhibitors is poor, and the selectivity of mDIVI-1, a widely used Drp1 inhibitor, has been questioned [63].

In this work, we evaluated whether two natural citrus-derived flavonoids could act as Drp1 inhibitors. Flavonoids are natural compounds present in fruits, vegetables, and seeds, with a broad-spectrum of beneficial activities against cardiovascular, neurodegenerative [64, 65], kidney diseases [66], and many other pathological settings including cancer [67–69]. These compounds have been also studied in the context of PC dyscrasias, as MGUS and MM, but the complexity in their mechanism of action has so far limited their clinical applications [70, 71].

In order to evaluate whether the two citrus-derived flavanones Hes and Nar could impact on mitochondrial dynamics, first we performed molecular docking studies exploiting the crystallographic structure of Drp1. Interestingly, both Hes and Nar were found to accommodate within the GTPase binding site of the protein with favorable energy values, anchoring Drp1 with binding modes and affinities comparable to previously reported synthetic inhibitors [40]. In line with an inhibitory effect on Drp1 fission activity, Hes and Nar increased fused mitochondria, as evidenced by TEM analyses, and reduced the levels of the S616-phosphorylated form of Drp1. Overall, these results indicate that Hes and Nar exert an antagonistic role on Drp1, prompting us to investigate the phenotypic and molecular changes promoted by both flavanones on MM cells.

Consistent with previous observations demonstrating that genetic or pharmacological inhibition of mitochondrial fission, or fusion promotion, can suppress the oxygen consumption rate of tumor cells [41], high resolution respirometry demonstrated that Hes and Nar reduced OXPHOS and ATP production, strengthening the mitochondrial activity of both compounds. Interestingly, Hes and Nar could reduce the clonogenic potential and the viability of MM cells, even resistant to standard-of-care treatments, like PIs, and such anti-MM effects were antagonized by Drp1 overexpression, again confirming a mitochondrial targeting, Drp1-dependent, activity of the two agents. Moreover, both Hes and Nar synergistically enhanced the anti-MM effects of PIs, without being cytotoxic to healthy PBMCs. Of note, Hes and Nar anti-MM effects were not antagonized by co-culture with patient-derived BMSCs, underscoring the potential ability of these two compounds to overcome the protective effects of the bone marrow microenvironment.

To shed further light on the molecular mechanisms underlying Hes and Nar-dependent effects in MM, we analyzed the transcriptomic perturbations following Hes and Nar treatment by the Ion AmpliSeq platform. Worthy of note, GO pathway analysis highlighted ER stress and intrinsic apoptosis as the top upregulated pathways, and we validated Hes- and Nar-dependent induction of ER stress and apoptosis-related genes by qRT-PCR and WB. These results indicate that both Hes and Nar, likely via Drp1 targeting, may induce metabolic changes with significant upregulation of the ER stress response ultimately leading to apoptosis.

Previous studies indicate that Drp1 contributes to metabolic plasticity, activating anabolic pathways in cancer cells [46]. The analysis of common DEGs between Hes and Nar evidenced a down-regulation of transcription factors, like c-MYC and SREBP1, involved in the transactivation of lipogenesis genes [47]. By using a reporter assay, we were able to demonstrate reduced triglycerides levels upon Hes and Nar treatment, along with a dramatic decrease in lipid droplets stored in MM cells, which was reverted by Drp1 overexpression. Since lipids are major building blocks but also act as signaling molecules in cancer cells, it is tempting to speculate that mitochondrial fission-reprogrammed lipid metabolism may take part to the growth of MM cells, which can be antagonized via Hes or Nar. Lipidomics analyses will be prospectively carried out to clarify the lipid classes affected by Hes and Nar in MM cells.

Both Hes and Nar are abundant in Tacle, a triploid citrus whose name name flavour recall the two parents’ cultivars: the Tarocco orange (*C. sinensis* L. Osbeck) and the Monreal Clementine (*C. clementina* Hort. ex Tan.) [72]. This innovative fruit was previously studied for its high antioxidant activity [73], likely contributing to its health-promoting effects, including lowering risk for chronic heart, vascular diseases, or metabolic disorders such as obesity and diabetes [45].Herein, we demonstrated that the extract of this fruit could produce the same anti-MM effects of the two flavanones. We observed in fact that Tacle extract could recapitulate both the phenotypic changes and the molecular perturbations triggered by Hes and Nar, and such effects were exploited in a proof-of-concept in vivo study using NOD/SCID mice xenografted with MM cells. Notably, Tacle treatment reduced the growth of MM xenografts, with potential on-target activity as demonstrated by reduced Drp1 protein levels, even in its fission-promoting S616-phosphorylated form, along with reduced expression of c-MYC and SREBP1, in resected tumor samples, without any evidence of toxicity towards mice.

Overall, our results suggest the possibility of using the flavanones herein investigated for targeting mitochondrial fission, especially exploiting their anti-MM activity. Follow up studies are mandatory for an in-depth investigation of the pharmacokinetics and the development of novel formulations based on Tacle, or its major flavanones, for cancer treatment. Moreover, given the physiological role played by mitochondrial dynamics in the context of immune and endothelial cells[8, 74, 75], and their dysregulation in different cancer types, including PC dyscrasias [76–79], future studies will be aimed at investigating whether Hes- and Nar-dependent Drp1 inhibition could also target aberrant angiogenesis and immune dysfunction acting on other cell populations of the MM bone marrow microenvironment.

## Conclusions

By targeting Drp1, Hes and Nar revert aberrant mitochondrial dynamics and lipid metabolism, triggering ER stress and apoptosis in MM. Taken together, these findings provide the first rationale to antagonize mitochondrial fission in cancer exploiting two natural flavanones highly abundant in Tacle citrus*.*

### Supplementary Information

**Additional file 1: Figure S1.** Hes and Nar content in Tacle^®^ extract after 7-week storage under controlled ambient conditions (1 ℃ and 90–95% RH). Values are expressed as means ± SD on three different measurements.** Figure S2.** Interaction of Hes and Nar with key amino acid residues of the Drp1 active site. Ligand, Structure, Pose, Binding Energy and Interactions (Hydrogen and hydrophobic bonds) of both Hes and Nar compounds are reported; docking score values are expressed in kcal/mol.** Figure S3.** WB analysis of MFN2 protein levels in AMO cells, 48h after treatment with increasing doses of Hes or Nar. Normalization was performed using GAPDH as loading control.** Figure S4.** Representative traces of respiration and O2 concentration in H929 cells, 48h after treatment with A) Hes (250µM) or (B) Nar (250µM). O2 flux per volume in the two chambers was measured simultaneously by Oroboros O2k Instrument, performing consecutive injections of Oligomycin (2µM), FCCP (0.5µM) and Antimycin A (2µM), according to SUIT 003 D009 protocol. Traces were analyzed by DatLab 7 software.** Figure S5.** Half-maximal inhibitory concentration (IC50) of Hes (left) and Nar (right) determined for AMO, AMO-BZB, H929, H929-BZB, and H929-CFZ cell lines. The IC50 were calculated using GraphPad Prism software from three independent experiments.** Figure S6.** FACS analysis of Annexin V/7-AAD stained AMO and AMO-BZB cells exposed for 48h to different concentrations of (A) Hes or (B) Nar. Representative dot plots of the data from an independent biological replicate (n=3) are shown.** Figure S7.** Fluorescence microscopy analysis of lipid droplets in AMO cells, 24hafter Hes (250µM) or Nar (250µM) treatment, labeled with BODIPY 493/503 probe. Representative images are reported (40x magnification). Histogram bars reported the number of lipid droplets ± SD in at least 100 cells from three different fields. *p<0.05.** Figure S8.** Representative traces of respiration and O2 concentration in H929 cells, 48h after treatment with Tacle (2mg/ml). O2 flux per volume in the two chambers was measured simultaneously by Oroboros O2k Instrument, performing consecutive injections of Oligomycin (2µM), FCCP (0.5µM) and Antimycin A (2µM), according to SUIT 003 D009 protocol. Traces were analyzed by DatLab 7 software.** Figure S9.** WB analysis of S616 p-Drp1 and Drp1 (A), ATF4, IRE1α (B), Caspase 3 (C) and DGAT2 (D) in lysates from AMO cells exposed for 48h to Tacle extract. GAPDH was used as protein loading control. E) Fluorescence microscopy analysis of lipid droplets in AMO cells, 24h after Tacle extract (2 mg/ml) exposure, labeled with BODIPY 493/503 probe. Representative images (40x magnification) are reported. Histogram bars represent the number of lipid droplets ± SD in at least 100 cells from three different fields. *p<0.05.** Figure S10.** A) Fluorescence microscopy analysis of lipid droplets in AMO cells stably expressing DNM1L, or the empty vector, labeled with LipidTOX Red probe, 24h after Hes (250µM), Nar (250µM) or Tacle extract (2 mg/ml) exposure. Representative images (100x magnification) are reported. Histogram bars reported the number of lipid droplets ± SD in at least 100 cells from three different fields. *p<0.05. B) Triglycerides measurement using Triglycerides Glo assay in DNM1L overexpressing AMO cells, 24h after Hes (250µM), Nar (250µM) or Tacle extract (2 mg/ml) exposure. Luminescence was evaluated at 48h. *p<0.05.** Figure S11.** H&E staining (20x magnification) of vital kidney, spleen, liver and heart tissue retrieved from mice, two weeks after treatments. Representative images are reported.

## Data Availability

Transcriptomic data are available through GEO accession number GSE253452. All the other data generated or analyzed during this study are included in this manuscript.

## Abbreviations

ACC: Acetyl-coa carboxylase
ACL: ATP-citrate lyase
BM: Bone marrow
BMMCs: Human bone marrow mononuclear cells
BMSCs: Human bone marrow stromal cells
BZB: Bortezomib
CFZ: Carfilzomib
CI: Combination index
CTG: Cell titer glo
DEG: Differential gene expression
Drp1: Dynamin-related protein 1
ER: Endoplasmic reticulum
FAS: Fatty acid synthase
FIS1: Fission 1 protein
GO: Gene ontology
Hes: Hesperetin
ISR: Integrated stress response
Mfn1: Mitofusin 1
Mfn2: Mitofusin 2
MFF: Mitochondrial fission factor
MM: Multiple Myeloma
MOM: Mitochondrial outer membrane
Nar: Naringenin
OCR: Oxygen consumption rate
OXPHOS: Oxidative phosphorylation
Opa1: Optic atrophy 1
PIs: Proteasome inhibitors
TEM: Transmission electron microscopy
