# Supplementary material for: Targeting of mitochondrial fission through natural flavanones elicits anti-myeloma activity
Source: J Transl Med. 2024 Feb 27;22:208. doi: 10.1186/s12967-024-05013-0 (PMC10898065; doi:10.1186/s12967-024-05013-0)

Supplementary Figure 1

Compound	Content (ppm)
Hes	210.7 ± 6.5
Nar	85.4 ± 5.4

Supplementary Figure 2

Binding energies for NAR and HES and key interactions with protein residues of Drp1

LIGAND	STRUCTURE	Binding Energy kcal/mol	INTERACTIONS				
			Hydrogen Bonds				Hydrophobic Interactions
			Residues	Distance (Å)		Donor-Hydrogen-Acceptor angle (°)	Residues
				H-A	D-A		
**NAR**	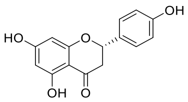	-7.4	Ser40	1.89	2.72	142.57	Lys216, Asn246
			Lys216	2.36	3.18	139.95	
			Asn246	2.29	3.20	152.91	
			Asn246	2.23	3.16	159.67	
			Gln249	2.69	3.59	152.46	
**HES**	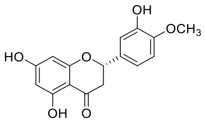	-7.6	Ser40	2.43	3.26	144.00	Asn246
			Lys216	2.29	3.15	146.04	
			Asp218	3.13	4.02	152.16	
			Asn246	2.06	2.99	156.22	
			Gln249	3.09	3.97	149.92	

Supplementary Figure 3

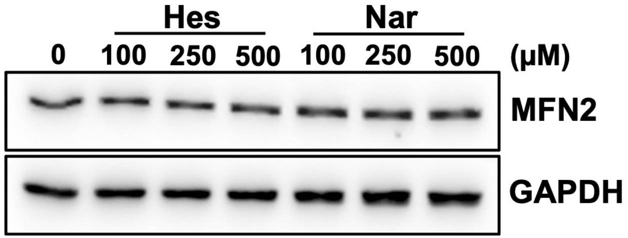

Supplementary Figure 4

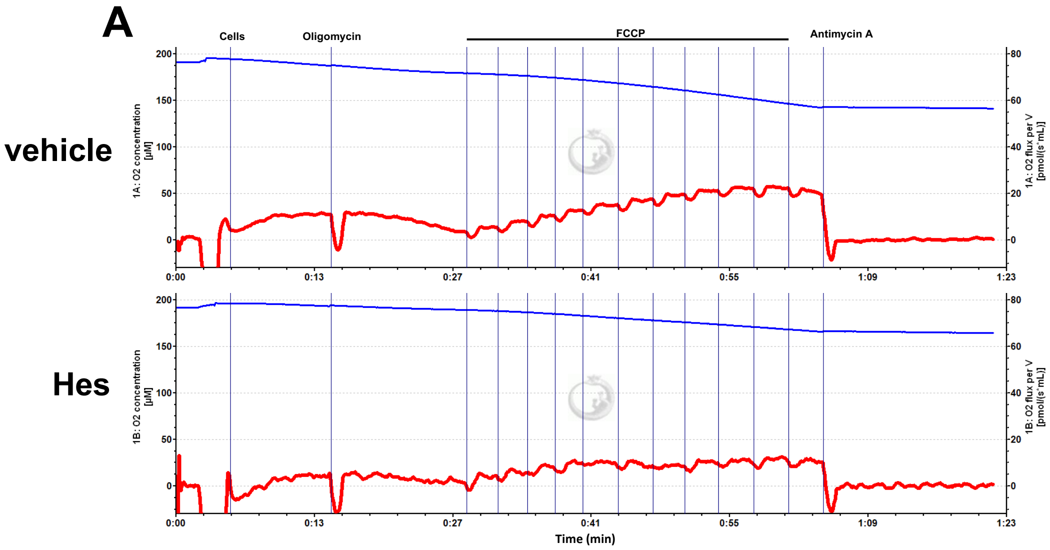

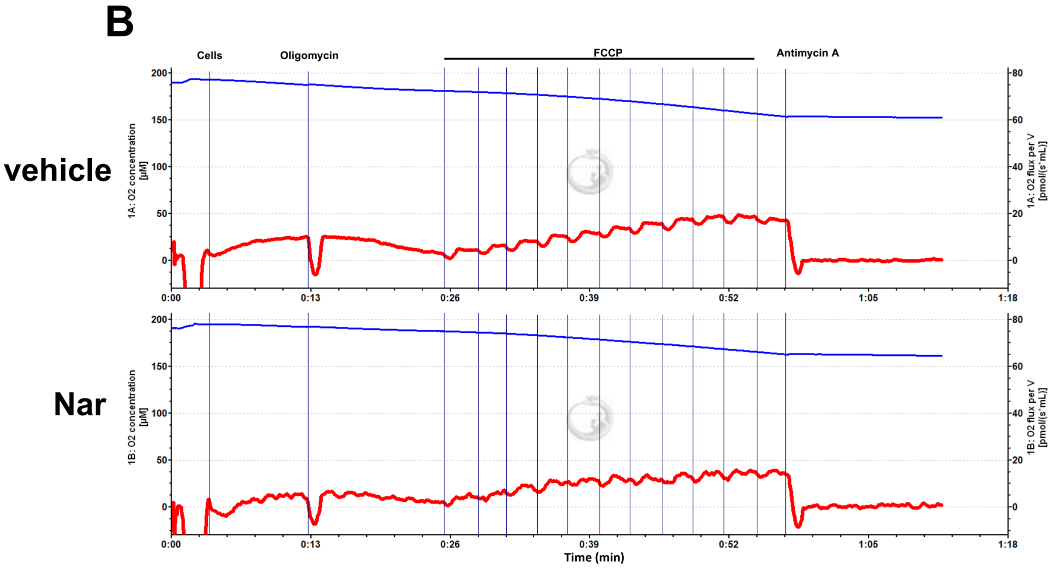

Supplementary Figure 5

A

MM cell lines	IC_{50} Hes
AMO	202 µM
AMO-BZB	386.6 µM
H929	194.1 µM
H929-BZB	361.9 µM
H929-CFZ	257.7 µM

B

MM cell lines	IC_{50} Nar
AMO	264.8 µM
AMO-BZB	352.8 µM
H929	263.4 µM
H929-BZB	273.3 µM
H929-CFZ	300.3 µM

Supplementary Figure 6

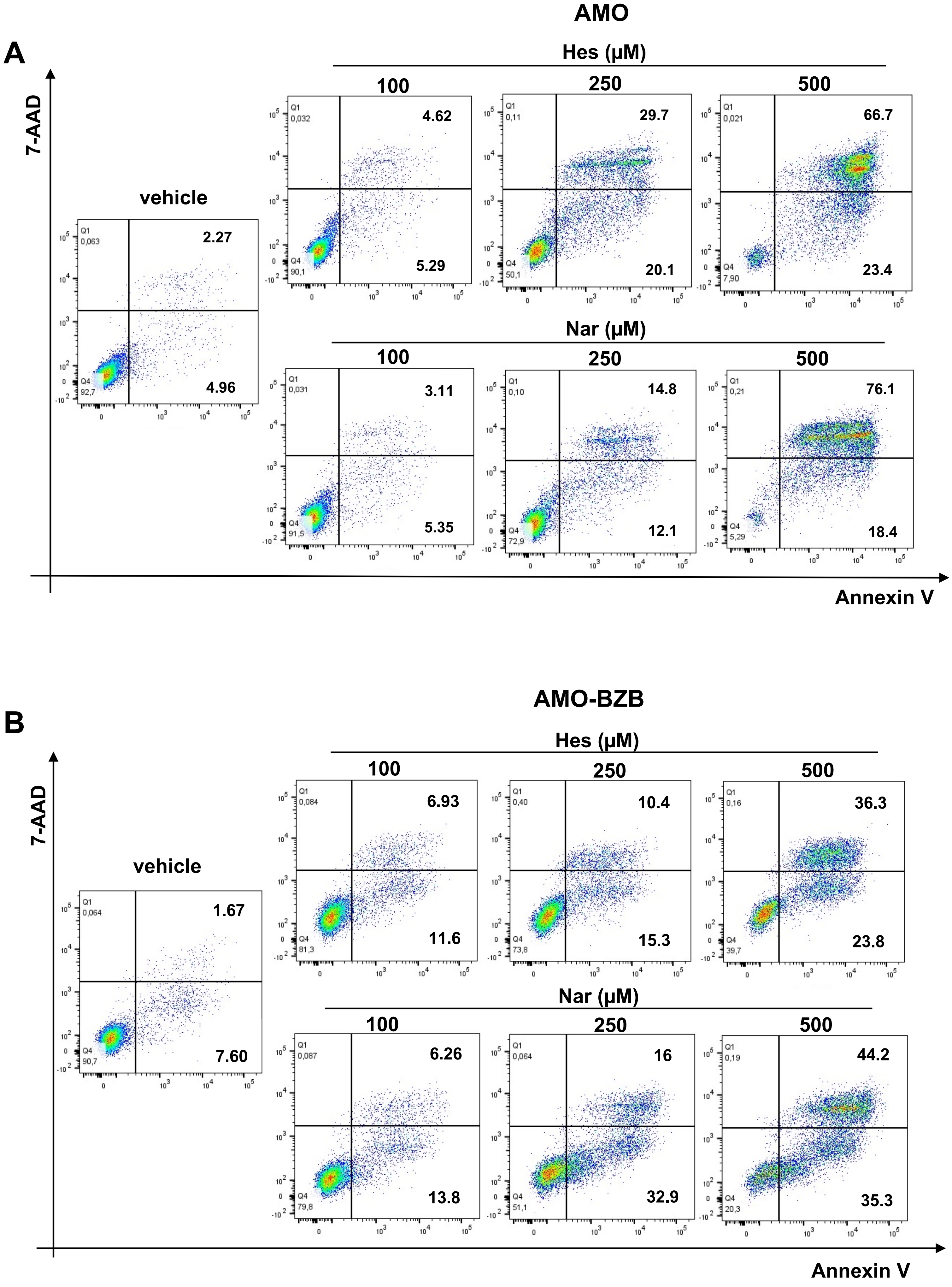

Supplementary Figure 7

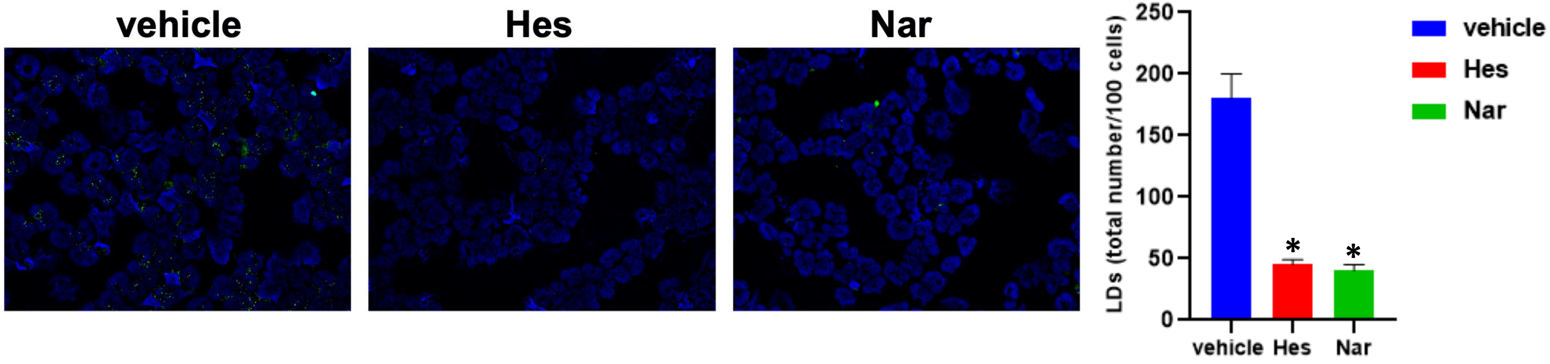

Supplementary Figure 8

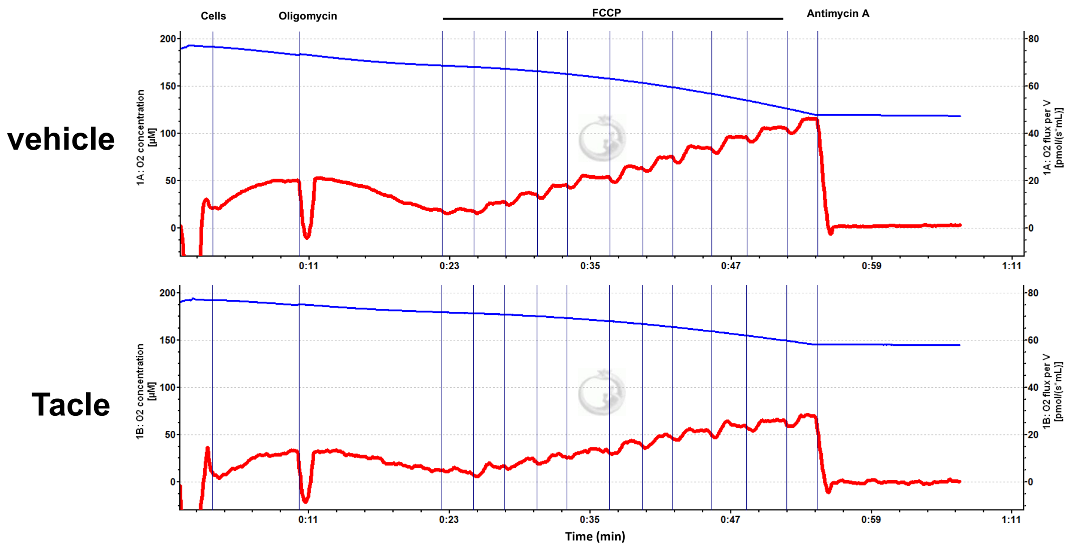

Supplementary Figure 9

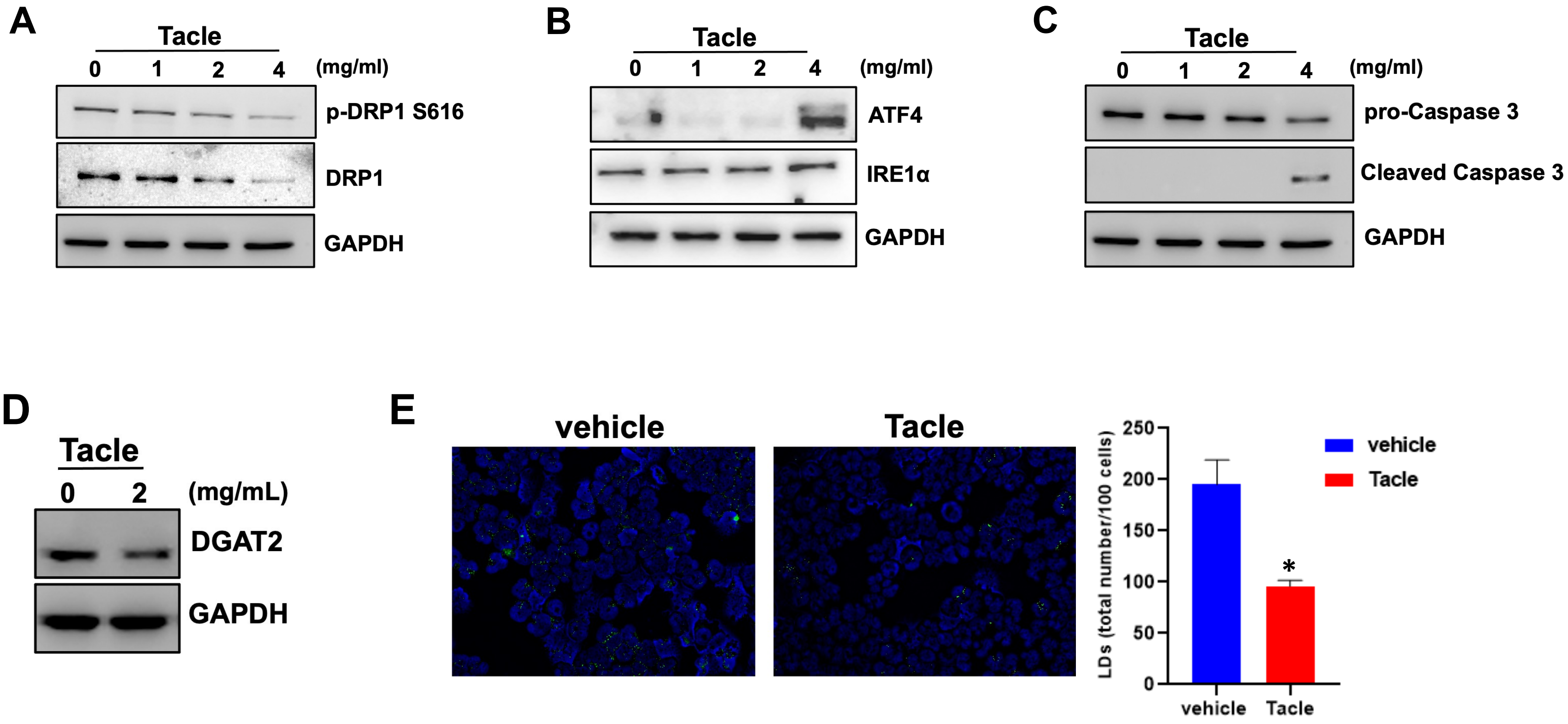

Supplementary Figure 10

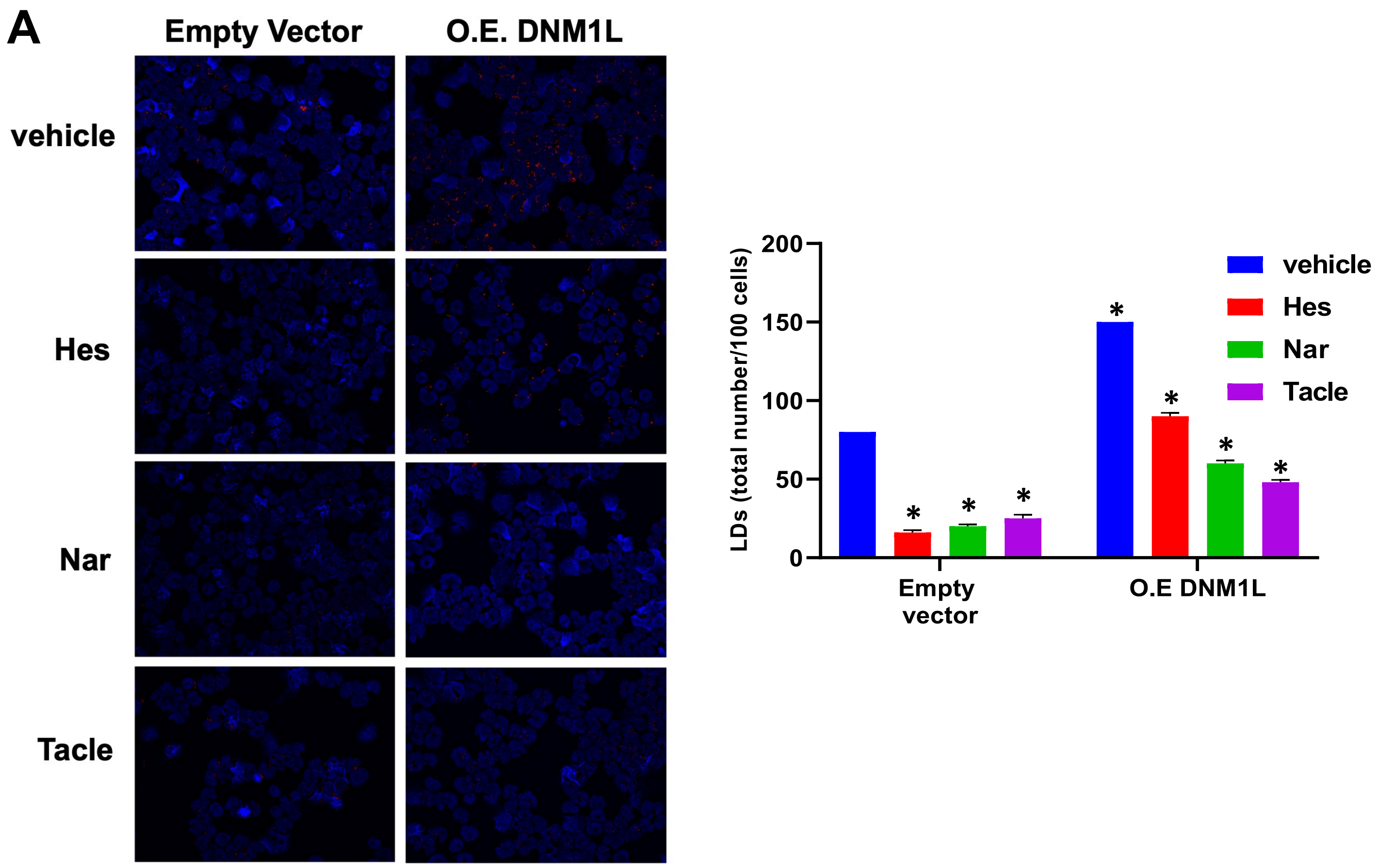

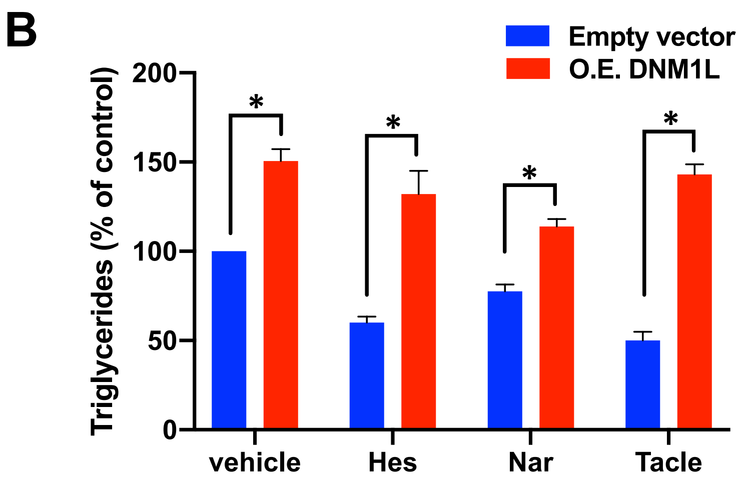

Supplementary Figure 11

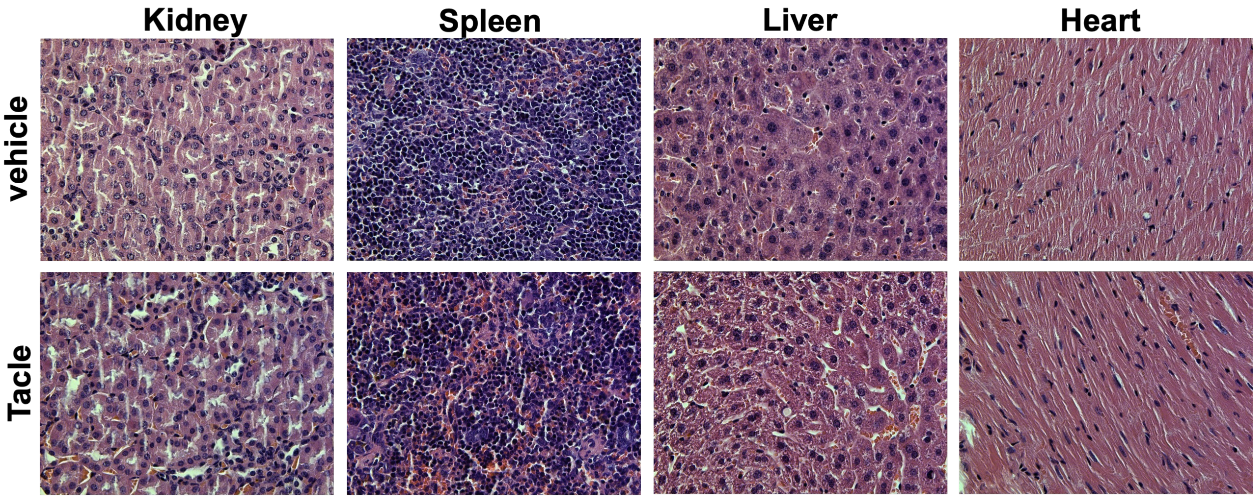

Supplement: Supplementary file 1 — Additional file 1: Figure S1. Hes and Nar content in Tacle® extract after 7-week storage under controlled ambient conditions (1 ℃ and 90–95% RH). Values are expressed as means ± SD on three different measurements. Figure S2. Interaction of Hes and Nar with key amino acid residues of the Drp1 active site. Ligand, Structure, Pose, Binding Energy and Interactions (Hydrogen and hydrophobic bonds) of both Hes and Nar compounds are reported; docking score values are expressed in kcal/mol. Figure S3. WB analysis of MFN2 protein levels in AMO cells, 48h after treatment with increasing doses of Hes or Nar. Normalization was performed using GAPDH as loading control. Figure S4. Representative traces of respiration and O2 concentration in H929 cells, 48h after treatment with A) Hes (250µM) or (B) Nar (250µM). O2 flux per volume in the two chambers was measured simultaneously by Oroboros O2k Instrument, performing consecutive injections of Oligomycin (2µM), FCCP (0.5µM) and Antimycin A (2µM), according to SUIT 003 D009 protocol. Traces were analyzed by DatLab 7 software. Figure S5. Half-maximal inhibitory concentration (IC50) of Hes (left) and Nar (right) determined for AMO, AMO-BZB, H929, H929-BZB, and H929-CFZ cell lines. The IC50 were calculated using GraphPad Prism software from three independent experiments. Figure S6. FACS analysis of Annexin V/7-AAD stained AMO and AMO-BZB cells exposed for 48h to different concentrations of (A) Hes or (B) Nar. Representative dot plots of the data from an independent biological replicate (n=3) are shown. Figure S7. Fluorescence microscopy analysis of lipid droplets in AMO cells, 24hafter Hes (250µM) or Nar (250µM) treatment, labeled with BODIPY 493/503 probe. Representative images are reported (40x magnification). Histogram bars reported the number of lipid droplets ± SD in at least 100 cells from three different fields. *p<0.05. Figure S8. Representative traces of respiration and O2 concentration in H929 cells, 48h after treatment with T [file 12967_2024_5013_MOESM1_ESM.pdf]
